# Social cognition, socioeconomic status and subjective well-being of Chinese migrant workers

**DOI:** 10.1038/s41598-024-56710-1

**Published:** 2024-03-19

**Authors:** Weichao Huang, Shipeng Su, Xiaoyu Sun

**Affiliations:** 1 School of Public Administration and Law, Fujian Agriculture and Forestry University, Fuzhou, 350002 China; 2 School of Rural Revitalization, Fujian Agriculture and Forestry University, Fuzhou, 350002 China; 3 School of Public Administration, Huazhong Agricultural University, Wuhan, 430070 China

**Keywords:** Migrant workers, Subjective well-being, Social cognition, Socioeconomic status, Intergenerational differences, Psychology, Health occupations

## Abstract

Subjective well-being is based on the unity of internal and external needs, as well as material and non-material needs. However, existing research lacks consideration of the impact of both objective material conditions and subjective psychological cognition on the subjective well-being of migrant workers. Thus, based on data from the 2017 China General Social Survey, this paper applies ordered logit models and OLS models to investigate the impact of social cognition and socioeconomic status on the subjective well-being of migrant workers and their intergenerational differences. The results indicate that: (1) Social cognition has a significant impact, and the impact of fairness perception is more pronounced than depression perception and class change perception; (2) among socioeconomic status, personal income did not have a significant effect as education level, car ownership and house property ownership; (3) there are intergenerational differences. The emotional state of the older generation is the most critical factor influencing their subjective well-being. In contrast, the new generation is more concerned with their feelings about future expectations. The older generation is more concerned with their house property ownership, while the increase in income, education and car ownership can significantly increase the subjective well-being of the new generation. For this reason, we believe that the Chinese government should gradually change the existing urban and rural management system to create a fair and just social environment; make migrant workers receive the same protection as urban residents and improve the income distribution mechanism; pay attention to the social security of the older generation of migrant workers and the development opportunities of the new generation of migrant workers and their ability to integrate into the city to improve their subjective well-being.

## Introduction

The course of human development is also the course of the pursuit of happiness. The report of the 19th National Congress of the Communist Party of China (CPC) points out that “the main contradiction in our society has been transformed into the contradiction between people's growing need for a better life and unbalanced and insufficient development.” The people's definition of happiness is no longer limited to solving the problem of food and clothing but includes more diverse and advanced content, such as satisfying spiritual needs and achieving all-around development of the individual body and mind. Since the reform and opening up, China's economy and society have developed rapidly, and a large number of migrant workers have flowed into cities and made great contributions to the modernization of the country. The relevant data from the National Bureau of Statistics show that the total number of migrant workers in 2023 is 297.53 million, an increase of 0.6% over the previous year. Among them, 176.58 million migrant workers went to cities, a rise of 2.7%^1^. As a disadvantaged group in the city, the subjective well-being of migrant workers is closely related to China's livelihood work. Improving the subjective well-being of migrant workers is an important part of realizing Chinese modernization. In order to meet the Chinese government’s goal that new urbanization should be people-centered and meet the people’s needs for a happier and higher-quality life. When studying the subjective well-being of migrant workers, their quality of life and psychological feelings should be comprehensively considered.

Well-being is a broad phenomenological category that includes people's emotional responses, domain life satisfaction, and comprehensive judgments of life satisfaction^2^. Therefore, there are three general expressions of Well-being in quantitative analysis, namely, Subjective Well-being^3^, Life Satisfaction^4^, and Ladder-of-life^5^, which are interchangeable and can be used as proxies for Well-being. These variables are interchangeable and can be used as proxies for well-being.

Numerous fields, including psychology, sociology, and economics, are active in subjective well-being research, the most prominent being economics research on the relationship between income and subjective well-being. Since the formulation of the “Easterlin Paradox” in 1974, numerous academics have examined the standard theories of contemporary economics to investigate the mechanism of the “happiness-income puzzle.” Studies that use absolute income as an entry point conclude that there is no significant correlation between average income and happiness levels^6,7^. From the perspective of adaptation theory, the increase in income will result in the psychological habit of automatic adaptation to high income, such that improving economic conditions has no significant effect on improving subjective well-being^8^. Meanwhile, studies based on Maslow's needs theory confirm the law of marginal diminution of residents' income on subjective well-being^9^. Studies examining the relationship between absolute income and subjective well-being from the perspective of income disparity mainly contain the following two views: First, the “expectation change theory,” which states that income difference can affect people's subjective well-being by altering their income expectations, including the “positive tunnel effect”^10–12^ and the “negative tunnel effect”^13–15^. Second, the “relative deprivation theory” states that economic discrepancy causes individuals to feel comparatively deprived throughout the comparison process, which in turn impairs their happiness experience^16^.

Inspired by foreign research, Chinese scholars have launched a series of research. Individual-level research has steadily shifted its focus from income to non-economic determinants like health^17^, marital quality^18^, employment status^19^, and psychological status^20^. The research on social features includes political issues like basic public services^21^, official corruption^22^, and government quality ^23^; social elements like social class^24^, social trust^25^, and social relationships^26^; and ecological environment like environmental pollution^27^. Beneficial research conducted by Chinese academics on the subjective well-being of urban and rural populations in China provides a new reference point for government policymakers seeking to reform development paradigms and increase national happiness. Despite these accomplishments, it is clear that the focus of domestic study remains on urban and rural residents and that research on the subjective well-being of migratory workers still needs to be strengthened.

In the framework of the new economic norm, industrial restructuring and social development have made it more difficult for migrant workers to experience subjective well-being. The subjective well-being of migrant workers who have left their hometowns is based on the unity of internal and external needs, material and immaterial needs, which not only derive from their subjective perception and self-evaluation of their lives but also depend on the improvement of their social status to satisfy their needs of belonging, respect, and self-fulfillment. To improve their subjective well-being, migrant workers must not only overcome the psychological challenge of being “marginalized in the city” and get psychological recognition and adaption but also assess if an increase in pay alone can meet their material demands^28^. In this setting, what are the novel aspects of the relationship between the social perceptions and socioeconomic status of migrant workers and their subjective well-being? How should policymakers modify their governance techniques to accommodate the increased needs of migrant workers? The solutions to these concerns require extensive empirical research based on data from microscopic studies of migrant workers. In this regard, this paper examines the logical relationship between social cognition, socioeconomic status, and subjective well-being using CGSS2017 research data, with the expectation that the study will serve as a reference for the formulation and execution of government policies in the new era.

Additional studies on discrimination perception^29^, depression perception^30^, fairness perception^31^, and class change perception^32^ demonstrate how migrant workers' impressions of society and life influence their subjective well-being experiences. And research on permanent assets such as house property ownership^33^ and automobile ownership^34^ demonstrates how the possessions owned by migrant workers can improve their subjective well-being by satisfying multi-level demands such as security, sociability, and respect.

By analyzing the existing research, we find that, first, psychological factors and research on the subjective well-being of migrant workers rarely involve social cognition; second, subjective well-being is a pleasant psychological experience or spiritual feelings, and its influencing factors must include two basic dimensions: subjective and objective^35^. Considering objective well-being conditions or individual cognitive levels alone is likely to cause model-setting bias due to omitting key variables. In addition, in the context of rapid urbanization and continuous changes in population structure, the heterogeneity of migrant workers has become increasingly prominent. As a new variable that cannot be ignored, intergenerational differences should be included in the analytical framework of migrant workers' subjective well-being.

Therefore, to supplement the research in this field, this study will focus on the following aspects: firstly, incorporating social cognition and socio-economic status into a unified framework, exploring the impact of internal subjective feelings and external objective factors on the subjective well-being of migrant workers; secondly, explore the intergenerational differences in subjective well-being among different generations of migrant workers; finally, address potential endogeneity issues in the model and improve the reliability of empirical analysis results.

The potential contribution of this study is to provide evidence through empirical analysis on the impact of social cognition on the subjective well-being of migrant workers in psychological factors; analyze whether the subjective well-being of migrant workers is influenced by their inner feelings or material conditions; further analyze the differences in the impact of intergenerational factors on the above two.

The remaining parts of this article are arranged as follows: The second part is theoretical analysis and research hypotheses; The third part introduces the data and methods; The fourth part is the estimation results and analysis, including benchmark regression results, intergenerational differences, and robustness tests; The fifth part discusses the limitations of this study and the research that should be extended on this basis; The final part is the conclusion and policy implications.

## Theoretical analysis and research hypotheses

### Social cognition and subjective well-being

Social cognition in sociology can be traced back to Durkheim, and he proposed a concept of collective representation that encompasses all elements of cognition present in the associative ties and group relations of social members. In subsequent studies, although scholars produced different insights on the analytical dimensions of social cognition^36^, they have reached a consensus on the basic connotation of social cognition, that is, social cognition includes the cognition of self, others, the surrounding environment, and social phenomena.

In social cognition, emotions, attitudes and social perception are the core themes. Regarding emotions, subjective well-being encompasses the individual's subjective evaluation of long-term emotions. It is self-evident that negative emotional experiences brought about by stress and depression lead to a decrease in subjective well-being^37^. Regarding social attitudes, as the economy grows, the pursuit of fairness and justice among the populace grows steadily. People's conceptions of social justice are evolving from “Not concerned about a shortage, but concerned about unequal distribution” to “Not concerned about scarcity, but concerned about unequal distribution, more concerned about unfair distribution.” The lower an individual's perception of social fairness, the lesser their motivation to pursue goals, which impacts their subjective well-being.

Regarding social perception, this study focuses on migrant workers' perception of their future class changes. Regarding the relationship between social class and subjective well-being, whether it is a sample study of transnational immigrants^38^ (p 34) or a specific analysis of internal immigrants in a certain country or region^39^, most of them have reached similar conclusions: social class identity and class upward mobility have a significant positive effect on subjective well-being. Based on this, put forward the hypothesis:

#### H1:

Positive social cognition has a positive and significant effect on the happiness experience of rural migrants.

It should be added that the subjective well-being of rural residents is more susceptible to changes in social class than urban residents. However, by working in metropolitan locations, migrant workers can enhance their income and hence improve their social status, reducing the influence of social class changes on the subjective well-being of the migratory worker group^40^. In terms of emotions, due to the opportunities for upward mobility and stronger social capital in migrant communities, migrant workers are less likely to experience negative emotions, and their mental health is in good condition. Similarly, migrant workers whose motives for migrating to work in cities are mainly focused on improving their economic conditions. During the working period, when institutional barriers posed by the household registration system significantly impeded the employment acquisition of migrant workers, the desire for fairness will have a more significant impact on subjective well-being than the perception of depression and class change. Based on this, put forward the hypothesis:

#### H2:

Among social perceptions, the effect of perception of fairness on subjective well-being was more pronounced compared to perception of depression and perception of class change.

### Socioeconomic status and subjective well-being

Socioeconomic status is a sociological concept that refers to the social position of an individual or group in the social structure defined by the social resources they possess. According to Maslow's theory of needs, the need for social status arises from the pursuit of belonging and self-actualization or belonging and self-actualization need to be realized through the acquisition of certain social status. In related studies, improving one's socioeconomic situation to achieve higher life satisfaction and subjective well-being has established a consensus in relevant studies. However, with rapid economic development, more and more research has indicated that socioeconomic position has a marginally diminishing effect on subjective well-being^41^, and some studies have found that persons who care about the materiality of wealth have lower subjective well-being^42^.

It should be noted that the effect of socioeconomic status on subjective well-being needs to consider differences in economic and cultural contexts. In a cross-country study, income and educational attainment predicted subjective well-being more for Chinese residents as subjects compared to Japan, Korea, and the United States^43^. Meanwhile, in a study on the relationship between income and happiness of migrant workers, Chinese scholars found that although there is an “Easterlin Paradox,” the remainder, after deducting the necessary expenses, contributes to the happiness level^44^. Therefore, when studying migrant workers, it is necessary to consider the specificity of the relationship between the acquisition of socioeconomic status and the subjective well-being of this group. Based on this, put forward the hypothesis:

#### H3:

Overall, the improvement of socioeconomic status contributes to the subjective well-being experience of the rural migrant group.

At the same time, we still need to pay attention to the different effects of the components of socioeconomic status on the subjective well-being of migrant workers. In the research on the impact of income on subjective well-being, it is generally believed that absolute income has an inverted U-shaped effect on subjective well-being. In contrast, relative income significantly affects subjective well-being more than absolute income^45^. However, the negative relationship between relative income and subjective well-being has been partially contested. Some research indicates that relative income also correlates favorably with subjective well-being^46^. This demonstrates that the mechanisms by which income affects subjective happiness are complicated and vary.

In contrast to income, the impact of education as a component of socioeconomic status on subjective well-being can not only enhance the social status of disadvantaged groups by reducing social exclusion but also translate into cultural capital, which can help individuals to obtain higher wage returns and more opportunities to leap up the social ladder in the future^47^. At the same time, under the influence of Chinese culture and historical traditions that have shaped the notion of educational rewards, social advancement and economic improvement are important expected outputs of education in the minds of the public: “There is a house of gold in books” reflects this folk “belief.” To this day, “reading changes one's fate” is still the typical popular perception of the value of reading. Thus, for Chinese residents, education implies the possibility of a leap up the social class. Specifically for the migrant worker group, the education they received before migration was more aimed at helping migrant workers understand urban life and adapt to the rhythm of urban life earlier, so the total effect of education on the well-being of migrant workers is greater than that of urban workers^48^. And the use of automobiles has enabled people to break through various spatial restrictions and further expand the “operating area” of the living world. On the one hand, automobile ownership helps to break spatial and temporal tensions and enhances their sense of empowerment in controlling their lives. On the other hand, automobiles meet social needs beyond material things, promoting subjective well-being through emotional communication and interpersonal interaction. Relevant studies show that rural residents have a higher sense of well-being when owning a car than urban residents^49^. For migrant workers, those who own cars tend to be able to build up their relationship networks in the city more quickly and efficiently, which can undoubtedly promote the integration of migrant workers into the urban life, reduce the psychological pressure brought about by the mobility of different places, and increase the sense of well-being. At the same time, the state of residence brought about by real estate reflects a relatively stable social relationship. The differentiation of the state of residence has become one of the most important contents of social stratification and social differentiation in Chinese cities^50^. Based on this, put forward the hypothesis:

#### H4:

Among socioeconomic status, education level and ownership of property and cars contribute more significantly to the subjective well-being of migrant workers than income.

### Intergenerational differences in the subjective well-being of migrant workers

Throughout the literature, existing research on intergenerational differences among migrant workers has advanced from investigating fundamental demographic factors to values, preferences, attitudes, and behaviors, as well as from the material to the spiritual level. Differences in the motivation, identity, and future development aspirations of older and younger migrant workers to work outside the home may also result in a variable logical relationship between their subjective well-being. The long-standing “hidden barriers” of China's urban–rural duality structure have misaligned the institutional identity of migrant workers with their actual identity. In addition, the identities of the younger and older generations of migrant workers have diverged due to their different life and employment histories. Older migrant workers seldom question their identities as farmers. Although they are constantly away from home, their psychological identity, economic ties, and social links to the countryside have not diminished.

On the contrary, the new generation of migrant workers generally lacks farming experience, blurring their institutional identity as “peasants.” In addition, the inculcation of urban culture continues to reduce their emotional identification with their hometowns, which directly affects their choice of future belonging, and integrating into the city and becoming a member of the city is the object pursued by new generation migrant workers. According to the social comparison model proposed by Falk and Markus (2004)^51^, the differences in identity and vernacular identity and future development expectations of the new and old generations of migrant workers may lead to differences in their choice of relative income reference groups and Chinese scholar Liu (2010)^52^, clarifies that the new generation of migrant workers has a stronger sense of relative deprivation because their chosen reference group is the workers in their place of work, while the reference group of the old generation of migrant workers is mostly the villagers in their old rural homes. Based on this, put forward the hypothesis:

#### H5:

 The older generation of migrant workers is more concerned about the influence of psychological aspects, i.e., social cognitive factors are more significant for the subjective well-being of the older generation of migrant workers.

#### H6:

The new generation of migrant workers is more inclined to integrate into the city, so improving socio-economic status can bring more subjective well-being.

## Data and methods

### Data source

The data used in this research comes from the 2017 Chinese General Social Survey, a large-scale nationwide survey designed to explore trends in social change. The China Survey and Data Center of the Renmin University of China carries out the survey. It adopts a four-level stratified sampling plan, covering multiple levels of individuals, families, communities and society. It is domestically recognized authoritative micro-survey data. Based on the characteristics of the sample required for this paper and the conventions of existing studies^53^, we screened with “whether engaged in non-agricultural industries” and “whether with agricultural household registration” and removed respondents with “undergraduate and above” education. Screen out 2030 samples of migrant workers that meet the needs of the research.

### Method

In the regression analysis, the ordered Logit model is often chosen to estimate the probability model of the dependent variable as an ordered variable. The model is set as follows:

1 $$ y_{i} = F\left( {\beta X_{i} + \varepsilon_{i} } \right) $$

In Eq. (1), ${y}_{i}$ is the dependent variable, i.e., subjective well-being. ${X}_{i}$ represents the independent variable (see table for details). ${\varepsilon }_{i}$ is the random disturbance term. F(.) is a nonlinear function, its concrete for

2 $$ F\left( {y_{i}^{*} } \right) = \left\{ {\begin{array}{*{20}c} {1,y_{i}^{*} < \mu_{1} } \\ { 2,\mu_{1} < y_{i}^{*} < \mu_{2} } \\ . \\ . \\ . \\ { j,y_{i}^{*} > \mu_{i - 1} } \\ \end{array} } \right. $$

In Eq. (2) ${\mu }_{1},{\mu }_{2}$, ……${\mu }_{i-1}$ are the cut points, all of which are parameters to be estimated. ${y}_{i}^{*}$ is the potential subjective well-being of the sample migrant workers, which is a latent variable due to the unobservability of this value. This latent variable satisfies the following conditions

3 $$ y_{i}^{*} = \beta X_{i} + \varepsilon_{i} $$

In the regression of the variables of interest, an ordered logit model was chosen based on the characteristics of the dependent variable. At the same time, the OLS model was selected to provide a more intuitive interpretation of the model fit's marginal effects and test the regression analysis's robustness without affecting the positive or negative values and significance of the coefficients of the variables.

### Selection of model variables

When using the Ologit model for regression, its proportional odds assumption needs to be considered. For this reason, we conducted the Wolfe Gould test on the Ologit regression model in this article. The results are as follows.

**Table:** 

	Model 1	Model 2	Model 3
Prob > chi^2^	0.055	0.064	0.086

According to the results, it can be seen that the O-Logit regression model used in this article meets the conditions of parallel regression assumption.

The purpose of this study is to explore the impact of social cognition and socioeconomic status on the subjective well-being of migrant workers and their intergenerational differences. Specific measures of various variables are as follows:

#### Dependent variable

This article selects the question “In general, do you think your life is happy?” in the questionnaire as a measure of subjective well-being. The answer options for this question include using a five-digit Likert scale, from “very unhappy” to “very happy”, and assigning values from 1 to 5 in turn. The average score of subjective well-being of migrant workers interviewed is 3.81, indicating that the subjective well-being of migrant workers is generally between "can't tell whether they are happy" or “relatively happy”.

#### Independent variables

The core independent variables in this paper include social cognition and socioeconomic status.

Social cognition: Individual perception and social perception comprise social cognition. Individual perception focuses mostly on an individual's evaluation of internal aspects, such as one's attitude and mood. In contrast, social perception encompasses an individual's feelings regarding social growth and evaluations of future situations. Therefore, based on the availability of data, this paper draws on the social perception research by Le^54^, and selects three indicators of depression perception, fairness perception and class change perception to measure the social cognition of migrant workers.

Socio-economic status: Socioeconomic status, as a multidimensional concept, reflects the stepwise inequality in the distribution of economic resources among different groups in society, emphasizing the economic status of individuals in society, often using economic income, education level and occupation as objective measures^55^. Notably, socioeconomic status is also determined by the ownership of fixed assets, such as the number of house properties and automobiles. In light of the unique characteristics of this group of migratory workers, this study considers personal income, education level, house property and automobile ownership to measure socioeconomic status.

#### Control variables

Based on existing studies on subjective well-being, we can learn that subjective well-being is also influenced by factors such as gender, education, age, marriage, number of children, and corresponding social security. In order to control other factors that may affect the subjective well-being of migrant workers and based on the premise of referring to previous papers, this paper selects gender, age, marital status, number of children, basic pension insurance, basic medical insurance, political outlook, labour contract signing status, and religious belief as control variables.

#### Instrumental variables

To overcome the possible endogeneity of the model, this paper choosing “Did you vote in the last village\neighborhood committee election?” as an instrumental variable. The reasons are: firstly, the familiarity status of community development history or voting experience reflects the social cognition of migrant workers to a certain extent, which satisfies the relevance requirement of instrumental variable; secondly, this variable describe migrant workers' recollection of relevant experiences or information in the past, which is not influenced by other factors at present, thus satisfying the exogeneity requirement of instrumental variables; thirdly, this variable has no direct effect on subjective well-being, but an indirect effect through social cognition. The specific definitions and descriptive statistics of the above four types of variables are shown in Table 1.

**Table 1:** Descriptive statistics.

Variable	Description	Mean	SD
SWB	5 points scale, Very unhappy = 1, very happy = 5	3.811	0.849
Depression perception	Rarely or never = 0, other = 1	0.661	0.474
Fair perception	Other = 0; fairer, perfectly fair = 1	0.440	0.496
Class change perception	Perception of class change differential in the next ten years Other = 0; Rising = 1	0.949	0.219
Logged annual income	Chinese yuan	10.36	1.670
Education level	Actual years of education	8.175	3.213
Automobile ownership	Own a automobile? No = 0, Yes = 1	0.276	0.447
House property	Number of house properties owned	0.677	0.626
Gender	Female = 0; Male = 1	0.586	0.493
Age	Age in 2016	44.299	13.983
Marital status	Unmarried or other = 0; Married = 1	0.849	0.357
Number of children	“How many children do you have?”	1.641	1.036
Basic pension insurance	No = 0; Yes = 1	0.606	0.489
Basic medical insurance	No = 0; Yes = 1	0.903	0.296
Political appearance	A member of the CPC? No = 0; Yes = 1	0.057	0.233
Employment contract	Sign an employment contract? No = 0; Yes = 1	0.159	0.365
Religious beliefs	Is there a religious affiliation? No = 0; Yes = 1	0.099	0.299
Migration distance	Current account registration. This city = 0;Other = 1	0.219	0.414
Voting experience	Vote in the last village election? No = 0; Yes = 1	0.450	0.498

## Results

### Analysis of the main effects of social cognition and socioeconomic status on subjective well-being

Table 2 lists the fitting results of all effective samples. Models 1–3 are the regression results obtained by fitting the ordered Logit model, respectively examining the influence of social cognition, socioeconomic status, and both on the subjective well-being of migrant workers. Model 4 is the result of the robustness test using the OLS model. According to the regression results of the model, the significant results of the core variables, such as social cognition and social status selected in this paper, are relatively consistent, indicating that the regression results have a certain degree of robustness.

**Table 2:** Ordered-logit and OLS estimates for social cognition and socioeconomic status on SWB.

Variables	Model 1	Model 2	Model 3	Model 4
	O-Logit	O-Logit	O-Logit	OLS
Depression perception	0.784***		0.749***	0.306***
	(0.098)		(0.098)	(0.039)
Fair Perception	0.950***		0.970***	0.379***
	(0.093)		(0.094)	(0.035)
Class change perception	0.172***		0.169***	0.062***
	(0.037)		(0.037)	(0.014)
Personal income		0.044	0.051*	0.022*
		(0.028)	(0.029)	(0.011)
Education level		0.032**	0.037**	0.012**
		(0.015)	(0.015)	(0.006)
Automobile ownership		0.531***	0.522***	0.180***
		(0.103)	(0.106)	(0.039)
House property ownership		0.190**	0.150*	0.059**
		(0.079)	(0.081)	(0.030)
Gender	− 0.156*	− 0.144	− 0.218**	-0.0968***
	(0.092)	(0.092)	(0.095)	(0.037)
Age	− 0.079***	− 0.010***	− 0.081***	-0.030***
	(0.020)	(0.021)	(0.021)	(0.008)
Age squared	0.001***	0.001***	0.001***	0.0004***
	(0.000)	(0.000)	(0.000)	(0.000)
Marital Status	0.986***	0.854***	0.862***	0.360***
	(0.145)	(0.149)	(0.149)	(0.062)
Number of children	0.015	0.017	0.027	0.004
	(0.053)	(0.053)	(0.053)	(0.021)
Basic pension insurance	0.232	0.0939	0.191	0.090
	(0.163)	(0.168)	(0.166)	(0.069)
Basic medical insurance	0.177*	0.155	0.099	0.055
	(0.101)	(0.101)	(0.101)	(0.0400)
Political appearance	0.443**	0.415**	0.373**	0.129**
	(0.174)	(0.180)	(0.176)	(0.061)
Employment contract	0.408***	0.410***	0.384***	0.137***
	(0.130)	(0.127)	(0.130)	(0.048)
Religious beliefs	0.134	0.179	0.190	0.0568
	(0.156)	(0.153)	(0.155)	(0.061)
Migration distance	− 0.217*	− 0.212*	− 0.264**	− 0.086**
	(0.111)	(0.111)	(0.113)	(0.044)
Provincial dummies	Yes	Yes	Yes	Yes
Observations	2030	2030	2030	2030

*P value < 0.1; **P value < 0.05; ***P value < 0.01.

#### The influence of social cognition on subjective well-being

The regression results in Table 2 indicate that, at the 1% significance level, perceptions of depression, fairness, and class change substantially impacted the subjective well-being of migrant workers in Models 1, 3, and 4. Therefore, hypothesis H1 is confirmed.

From a psychological perspective, we can understand that people with a positive emotional state can face life's challenges and failures with greater ease. This positive emotional experience can assist migrant workers in adapting to the “urban peripheral” mentality they encounter when living and working in the city, thereby enhancing their subjective happiness. In fairness perception, social comparison theory suggests that the nature and level of subjective well-being are frequently the consequence of social comparison^56^ and that the quality of this result depends on the direction and content of social comparison. For the group of migrant workers employed in different places in urban work, the reference object of comparison increases the group of urban residents. However, their treatment is completely different from urban residents, so their sense of relative deprivation is stronger. Therefore, the extent to which migrant workers are treated fairly, especially in terms of income, children's education, and intergenerational mobility, significantly impacts their subjective well-being. At the same time, the perception of class change also shows a significant positive effect on subjective well-being. This indicates that respondents' perceptions of future class changes have a goal-directed effect, enabling migrant workers to perceive and plan for their future. When individuals can focus on the longer-term future and perceive time and its value, they are better able to manage their time and are thus more motivated to achieve their goals. This motivation translates into expectations for the future, ultimately enhancing migrant workers' subjective experience of well-being. In the fitting results of social perceptions on migrant workers' subjective well-being, it can also be observed that the regression coefficients of perceptions of fairness are 0.950, 0.970, and 0.379, respectively, and the corresponding regression coefficients of perceptions of depression and class change are 0.784, 0.749, and 0.306 versus 0.172, 0.169, and 0.062. It can be inferred that fairness judgments have a higher impact on the subjective well-being of migrant workers than perceptions of depression and class change. Therefore, hypothesis H2 is verified.

The reason for this is that the initial intention of migrant workers to seek employment is typically strongly tied to family support, with the obvious motivation of economic demands. However, the institutional discrimination between urban and rural areas hinders their chances of finding suitable jobs. The gap between their expectations and reality leads to lower subjective happiness perceptions. At the same time, when the migrant population enters the city to work and live, their reference group becomes urban residents rather than community residents before mobility. This strong sense of material frustration makes them dissatisfied with their current living condition. Therefore, the desire for fairness will more obviously affect the subjective well-being of migrants.

#### The effect of socioeconomic status on subjective well-being

In the regression results in Table 2, the impact of personal income on the subjective well-being of migrant workers only shows a positive significance level of 10% in Model 4. However, neither Model 2 nor Model 3 shows a significant impact. The impact of education level, house property and automobile ownership on subjective well-being in models 2–4 are all positive and statistically significant at the 5% level. This demonstrates that the impact of socioeconomic status on the subjective well-being of migrant workers generally has a significant positive impact, and the impact of income on the subjective well-being of migrant workers is not obvious compared with the other three types of variables. Therefore, hypotheses H3 and H4 are verified.

The reason is that from the perspective of the “positive tunnel effect” in terms of income, people in a congested two-lane tunnel find that the cars in the next lane start to move forward, even though the lane they are in is congested, but still generate a sense of pleasure because of the optimistic expectation of getting out of the congestion, which also predicts a widening income gap. It can bring optimistic income expectations and thus increase one's subjective well-being^9^. However, it should be noted that if, after a long time, people find that only the lane next to them has been cleared, and their lane remains congested, the optimistic expectations will disappear and be replaced by dissatisfaction, anger, and even illegal lane changes. This is also known as “Negative Tunneling.” Although migrant workers' income climbed by 25.5% between 2012 and 2016, it is primarily utilized to pay for daily necessities. Compared to urban inhabitants, it is evident that the income growth of migrant workers does not result in a considerable increase in subjective well-being. In this case, it is not the family income that affects the subjective well-being of migrant workers, but the remaining part after deducting the necessary living expenses, that is, the amount of development expenditure, has a significant positive impact on their subjective well-being.

In contrast, education generates both consumer and production values for its beneficiaries. On the one hand, consumer value refers to the non-monetary return of education, which indicates the direct effect of features of consumer goods^57^. Education as a consumer commodity can boost an individual's physical and mental happiness, increasing their utility or subjective well-being. Productive value, on the other hand, reflects the indirect effect of education on subjective well-being. It refers to the indirect effect of education on subjective well-being by enhancing people's wage income or earning capacity.

In terms of house property, people are also concerned about the stability of life while pursuing income. In traditional Chinese thought, the house is the place to avoid danger and the space for social life. Owning a house property guarantees the life and work of migrant workers. Secondly, the asset attributes of house property are also an important factor. With the rising prices, house property is in a state of constant appreciation.

Moreover, house property is essential to the naturalization of migrant workers. It is intimately associated with the education of the children of migrant workers, access to public resources, and community integration. Therefore, both self-built and self-purchased residences can have a significant “happiness effect” on migrant workers. The influence of housing property on the subjective well-being of migrant workers is not limited to the provision of housing consumption flow; it also increases residents' subjective well-being by easing liquidity limitations and decreasing preventative savings.

It is also important to note that automobile ownership also contributes to the subjective well-being of migrant workers. First, as a commodity and a means of transportation, the automobile provides utility value and fulfills the need for transportation. Second, the increased radius of activity space and the breaking of fixed schedule constraints create a sense of psychological security. Owning automobiles helps them alleviate the spatial and temporal tension in modern society, maintain their existing social relationships, and enhance their current social capital by constructing opportunities for people to be socially present and increasing the probability of their social interactions. In other words, migrant workers who own automobiles tend to build their relationship networks in the city more quickly and efficiently, which undoubtedly promotes the integration of migrant workers into urban life, reduces the psychological strain caused t by foreign mobility, and enhances their subjective sense of well-being.

### Analysis of intergenerational differences in the effects of social cognition and social status on subjective well-being

Numerous studies on age differences in subjective well-being have revealed that, despite declining health and income levels with age, older adults' subjective well-being remains stable or even tends to increase. This phenomenon is known as the well-being paradox of the aging process. To maintain a sense of control and shield themselves from the shocks brought on by aging, older persons frequently pick a downward social comparison strategy during the social comparison process, according to research on this contradiction. Therefore, this study also investigates if the intergenerational inequalities between the older and younger generations of migrant workers become a factor affecting their subjective well-being due to high economic development and the marginally diminishing effect of income on subjective well-being.

In the regression results in Table 3, the subjective well-being of older generation migrant workers is significantly influenced by depression perception and fairness perception in terms of social perception, both of which are significant at the 1% level. In contrast, the variables in socioeconomic status only have a positive and significant effect on SWB in terms of house property at the 10% level, so hypothesis H5 is verified. The regression results of the new generation of migrant workers not only in socioeconomic status on subjective well-being but also personal income, education level and car status show positive significance at least 10% level, so hypothesis H6 is verified.

**Table 3:** Ordered-logit and OLS estimates for intergenerational differences.

Variables	New generation of migrant workers		Older generation of migrant workers	
	Model 1	Model 2	Model 3	Model 4
	OLS	O-Logit	OLS	O-Logit
Depression perception	0.299***	0.774***	0.311***	0.734***
	(0.070)	(0.169)	(0.048)	(0.123)
Fairness perception	0.448***	1.127***	0.363***	0.933***
	(0.061)	(0.164)	(0.042)	(0.115)
Class change perception	0.069***	0.174***	0.037	0.112*
	(0.018)	(0.049)	(0.022)	(0.054)
Personal income	0.039*	0.111*	0.008	0.010
	(0.022)	(0.062)	(0.013)	(0.032)
Education level	0.021*	0.050**	0.004	0.011
	(0.011)	(0.0239)	(0.007)	(0.02)
Car ownership	0.237***	0.651***	0.069	0.247
	(0.046)	(0.130)	(0.072)	(0.183)
House property ownership	0.013	− 0.001	0.064*	0.175*
	(0.062)	(0.157)	(0.035)	(0.095)
Marital status	0.346***	0.829***	0.264***	0.588***
	(0.098)	(0.236)	(0.0810)	(0.193)
Number of children	− 0.003	0.021	0.043*	0.127**
	(0.050)	(0.124)	(0.022)	(0.057)
Political appearance	0.035	− 0.043	0.201***	0.596***
	(0.117)	(0.343)	(0.068)	(0.197)
Employment contract	0.142*	0.323*	0.106*	0.363**
	(0.076)	(0.196)	(0.064)	(0.179)
Migration distance	− 0.076	− 0.233	− 0.120**	− 0.367**
	(0.070)	(0.177)	(0.059)	(0.154)
Other variables	Control	Control	Control	Control
Observations	670	670	1360	1360

*P value < 0.1; **P value < 0.05; ***P value < 0.01.

Intergenerational differences in the influence of social cognition-related variables on the subjective well-being of migrant workers are mainly manifested in the perception of class change. It is important to pay attention to the fact that the old and new generations of migrant workers are not at the same stage of social transformation, which also leads to differences in the logical relationship between the two at the level of social cognition and subjective well-being.

Specifically, the motivation of the older generation of migrant workers to work is summarized as a passive choice based on “survival rationality” and is mainly economical. While the new generation of migrant workers has diversified the purpose of going out to work from simply “earning money,” they consider earning money while pursuing an urban lifestyle, with the characteristics of economic and lifestyle coexisting.

Compared with the older generation of migrant workers, the new generation of migrant workers tends to see going out as a process of accumulating human capital and social capital and using it to transform their institutional identity**.** Therefore, the new generation of migrant workers pays attention to wages and improving their skills, the realization of rights and the long-term development of the future. Therefore, in the regression results in Table 3, we can see that the perception of class change has a significant impact on the subjective. There is a significant positive effect on subjective well-being, but not on the older generation of migrant workers.

There are also significant differences between the two in terms of socioeconomic status. First of all, only house property owner has a positive impact on the subjective well-being of the older generation of migrant workers. Personal income, education level, and car ownership all positively and significantly impact the subjective well-being of the new generation of migrant workers.

Although social perceptions and socio-economic status influence the subjective well-being of both generations of migrant workers, due to the differences in social motivation between the elderly and the young, the older generation's subjective well-being is less influenced by material-economic factors such as work and income. In contrast, emotional-psychological factors such as their ability and social relationships have a greater impact on subjective well-being. Young migrant workers pay more attention to materialistic economic status, so improving self and subjective well-being in economic comparison is easier. In the traditional concept, the prerequisite for providing for the elderly is “having a place to live.” Therefore, owning real estate has a decisive impact on improving the subjective well-being of older migrant workers. At the same time, the notion of old age being provided for is manifested in the older generation of migrant workers as child support, so the number of children significantly positively impacts the subjective well-being of the older generation of migrant workers.

For the new generation of migrant workers, whose motivation for working has changed, personal income and educational attainment are important influences on the subjective well-being of the new generation of migrant workers. For the new generation of migrant workers, whose social relationship formation is at a critical stage, automobile ownership helps them integrate into urban life more quickly, both in terms of reducing social tensions in time and space and in terms of symbolic solidarity and convergence of consumption that automobile ownership itself provides. Therefore, automobile ownership greatly improves the subjective well-being of the new generation of migrant workers.

Overall, the older generation of migratory workers gradually withdraws from the labor market as they age, and their focus switches from earning a living to retirement. Therefore, changes in retirement-related characteristics are more likely to affect the subjective well-being of this group. However, a shift in the desire of the new generation of migrant workers to work causes this group to place a greater emphasis on their materialistic economic status.

### Treatment of endogeneity of social cognitive variables

Since social cognition and subjective well-being are subjective variables, there may be some similar psychological mechanism of action, leading to reverse causality. Higher subjective well-being brings positive social cognition, while non-social cognition affects subjective well-being. Or there may be highly unobserved omitted variables that simultaneously affect social cognition and subjective well-being, resulting in biased estimates of social cognition's effect on subjective well-being.

To solve the possible endogenous problem of social cognition, this paper draws on the endogenous treatment of social cognition by Lu et al.^58^ to generate a new dummy variable: when the three variables measuring social cognition simultaneously take a value of 1, the dummy variable is assigned a value of 1 and named “positive cognition”, and use “whether there is voting experience” as an instrumental variable of positive cognition for endogenous testing and analysis.

In this paper, two-stage least squares (2SLS) were used for parameter estimation, and weak instrumental variable test and endogeneity test were used to determine the validity of the instrumental variables, exogeneity and endogeneity of the variable “positive cognition.” Table 4 displays the regression results.

**Table 4:** TSLS regression results of the effect of social cognition on the SWB of migrant workers.

	2SLS	2SLS	O-Logit	OLS
	Phase I Dependent variable: positive cognition	Phase II Dependent variable: SWB		
Voting experience	0.107***	–		
	(0.022)			
Positive perception	–	1.540***	1.051***	0.422***
		(0.432)	(0.101)	(0.034)
Adjusted R^2^	0.030	0.054		0.118

The first-stage regression results indicate that “voting experience” significantly impacts migrant workers' positive attitudes. The F-statistic of the joint significance test is 19.62, which is greater than 10, indicating that there is no weak instrumental variable; the *p*-values of the Sargan test and Basmann test are more significant than 0.01, indicating that the original hypothesis that the instrumental variable meets the condition of exogeneity cannot be rejected at the 1% statistical level; the p-value of the endogeneity Hausman test is 0.0023, which cannot overturn the original hypothesis that positive cognition is an exogenous variable.

According to the econometric theory, the ordered Logit and OLS models are more effective than the 2SLS models under the condition of no endogeneity. From the regression results of the ordered Logit model and OLS model, positive cognition has a significant positive effect on the subjective well-being of migrant workers, which again confirms the robustness of the results of the empirical analysis in this paper.

## Discussion

Social perception and socioeconomic status are important windows into the subjective well-being of migrant workers. Through this window, we can observe that: 1. In the midst of social change, both internal subjective feelings and external objective conditions have an important impact on the subjective well-being of migrant workers, and the degree of impact varies; 2. In the pursuit of happiness, there are obvious generational differences in the internal feelings and real needs of migrant workers of different generations, which mainly come from the different motivations for them to work.

As mentioned above, subjective well-being is a pleasant psychological experience or spiritual feeling, and its influencing factors must include two basic dimensions, subjective and objective. The analysis in this paper is only the beginning, and there are still many deficiencies that need to be further expanded in future research.

First, “Do you think your life is happy?” only measures one aspect of subjective well-being, namely positive emotions or affective well-being. The broader concept of subjective well-being includes other components, such as life satisfaction and purpose.

Second, future research must discuss in depth the impact of psychological factors on migrant workers’ subjective well-being. Both psychological changes in urban integration and research on reintegration into rural society are topics worthy of further discussion.

Third, future research needs to pay more attention to the impact of migrant worker group heterogeneity on subjective well-being. This article only considers the age factor. The heterogeneity of migrant workers should also consider the influence of gender, class, ethnic group, and region. Future research is necessary to theoretically explain and empirically test the mechanism by which migrant worker group heterogeneity affects subjective well-being from a spatial or regional perspective. All in all, these related issues still need to be further studied.

## Conclusions and policy implications

This paper uses the 2017 China General Social Survey data to analyze the impact of social cognition and socioeconomic status on the subjective well-being of migrant workers and their intergenerational differences. The following conclusions and corresponding policy implications are drawn:

### Conclusions

The results found that: (1) Social cognition as measured by depression, fairness and class change had a significant effect on the subjective well-being of migrant workers, with fairness having a more pronounced effect than the other two. This is similar to the findings of Htay's study on Malaysian migrant workers^59^ that migrant workers are more prone to mental health problems due to migration, separation from family, discrimination and social exclusion, which in turn affects their subjective well-being perception. Meanwhile, in the perception of fairness section, we are also concerned that although migrant workers achieve upward mobility from rural to urban areas, such mobility is highly vulnerable to the perception of fairness, and Hadjar and Samuel's study even found that upward mobility may reduce subjective well-being when the level of social inequality is high^60^. (2) Among the socio-economic statuses measured by personal income, education level, car ownership and house ownership, all the other socio-economic statuses have a significant positive effect except income, which has no significant effect on the subjective well-being of migrant workers. This suggests that with institutional change and social transformation, the subjective well-being of migrant workers is affected by various socio-economic statuses, and the weight of income's influence on subjective well-being decreases due to the diversification of work purposes. This conclusion is consistent with Zhang Lisi and Zhang Ziwei's conclusion after analyzing the CGSS data from 2010 to 2015, that is, subjective well-being increases and then decreases with the widening of the income gap, and that residents' tolerance of the income gap is declining^61^. (3) There are intergenerational differences between social cognition and socioeconomic status in migrant workers' subjective well-being. The emotional state of the older generation of migrant workers is the most influential factor on their subjective well-being, while the new generation of migrant workers is more concerned with the feeling of future expectations; the older generation of migrant workers is more concerned with the house property ownership, while the increase in income, education and automobile ownership can significantly improve the subjective well-being of the new generation of migrant workers.

### Policy implications

Firstly, it should be noted that the existence of urban–rural segmentation marked by the registered residence system is the source of negative emotions in the social comparison between migrant workers and urban residents. Therefore, the existing urban and rural management system should be gradually changed, and all social members and social groups should be encouraged to jointly create a fair and just social environment. At the same time, positive guidance should be provided to migrant workers in the process of working and living in cities, promoting the formation of reasonable psychological expectations and a good psychological state among migrant workers.

Secondly, according to neoclassical economic theory, the reason why immigrants are willing to migrate is to pursue higher economic benefits than their place of origin. The higher the socio-economic status of migrant workers, the more benefits they gain in the city. For migrant workers, extending their education years is not realistic. We should focus on improving their income and professional status, and vocational training is an important way. Therefore, it is necessary to continuously increase vocational training for migrant workers and improve the quality of vocational training.

Thirdly, we can understand that the older generation of migrant workers are gradually withdrawing from the labor market, so we should pay attention to their social security level. For the new generation of migrant workers, more development opportunities and greater development space should be created, and the integration of the new generation of migrant workers into the urban environment should be optimized to promote their sense of happiness.

## Data Availability

The data set used in this study was provided by the Center for Social Science Survey at Renmin University of China, and raw data can be applied via official email (cgss@ruc.edu.cn). The Stata code used for the paper is available upon request from the authors.
